# Efficacy and Safety of Dolutegravir Plus Emtricitabine vs Combined Antiretroviral Therapy for the Maintenance of HIV Suppression: Results Through Week 144 of the SIMPL’HIV Trial

**DOI:** 10.1093/ofid/ofae618

**Published:** 2024-10-16

**Authors:** Annalisa Marinosci, Delphine Sculier, Gilles Wandeler, Sabine Yerly, Marcel Stoeckle, Enos Bernasconi, Dominique L Braun, Pietro Vernazza, Matthias Cavassini, Laurent Decosterd, Huldrych F Günthard, Patrick Schmid, Andreas Limacher, Mattia Branca, Alexandra Calmy, the Swiss HIV Cohort Study, Rosemary Sudan, Charlotte Barbieux, Tamara Da Silva, Beatrice Bernasconi-Meyer, Karin J Metzner

**Affiliations:** HIV/AIDS Unit, Division of Infectious Diseases, Geneva University Hospitals, and the University of Geneva Faculty of Medicine, Geneva, Switzerland; HIV/AIDS Unit, Division of Infectious Diseases, Geneva University Hospitals, and the University of Geneva Faculty of Medicine, Geneva, Switzerland; Private Practice Office, Geneva, Switzerland; Division of Infectious Diseases, Bern University Hospital, University of Bern, Bern, Switzerland; Institute of Social and Preventive Medicine, University of Bern, Bern, Switzerland; Laboratory of Virology, Geneva University Hospitals, Geneva, Switzerland; Division of Infectious Diseases and Hospital Epidemiology, University Hospital of Basel, University of Basel, Basel, Switzerland; Division of Infectious Diseases, Ente Ospedaliero Cantonale, Lugano, Switzerland; University of Geneva and University of Southern Switzerland, Lugano, Switzerland; Department of Infectious Diseases and Hospital Epidemiology, University Hospital of Zurich, University of Zurich, Zurich, Switzerland; Institute of Medical Virology, University of Zurich, Zurich, Switzerland; Division of Infectious Diseases and Hospital Epidemiology, Kantonspital St. Gallen, St. Gall, Switzerland; Division of Infectious Diseases, University Hospital of Lausanne, Lausanne, Switzerland; Laboratory of Clinical Pharmacology, Department of Laboratory Medicine and Pathology, Lausanne University Hospital and University of Lausanne, Lausanne, Switzerland; Department of Infectious Diseases and Hospital Epidemiology, University Hospital of Zurich, University of Zurich, Zurich, Switzerland; Institute of Medical Virology, University of Zurich, Zurich, Switzerland; Division of Infectious Diseases and Hospital Epidemiology, Kantonspital St. Gallen, St. Gall, Switzerland; CTU Bern, University of Bern, Bern, Switzerland; CTU Bern, University of Bern, Bern, Switzerland; HIV/AIDS Unit, Division of Infectious Diseases, Geneva University Hospitals, and the University of Geneva Faculty of Medicine, Geneva, Switzerland

**Keywords:** dolutegravir + emtricitabine, HIV-1 dual therapy, maintenance treatment, patient satisfaction, quality of life

## Abstract

The SIMPL’HIV study investigated whether switching to dolutegravir (DTG) + emtricitabine (FTC) was noninferior to continuing combined antiretroviral therapy for maintaining HIV-1 suppression at 144 weeks. The study demonstrated that viral suppression, CD4 gains, adverse events, quality of life, and patient satisfaction were comparable between groups, confirming DTG + FTC’s safety and efficacy for long-term management of HIV-1 infection.

In recent years, dolutegravir-based dual therapy has become a viable alternative to triple combination antiretroviral therapy (cART). Approved by the Food and Drug Administration (FDA) [1] and the 2021 European AIDS Clinical Society (EACS) guidelines [2], it is recommended for both initial and maintenance therapy in adults with HIV-1. Dual therapy can reduce toxicity and costs and improve tolerability, adherence, and quality of life [3].

While some 2-drug regimens have not met efficacy or safety expectations [4–6], others have shown virological noninferiority to 3-drug therapies, with comparable CD4 cell count reconstitution and favorable safety profiles [7–28]. The efficacy and safety of dolutegravir plus lamivudine (DTG/3TC) have been validated in large clinical trials involving treatment-naïve adults [7, 8]. Four clinical trials have demonstrated that dual therapy with DTG plus 3TC or emtricitabine (FTC) is noninferior to triple therapy for maintaining viral suppression up to week 48 [12, 28–32], with the TANGO study confirming the noninferiority and safety up to 144 weeks [11].

In the SIMPL’HIV study, switching to DTG + FTC was noninferior to continuing cART, with around 91% of participants maintaining HIV-1 RNA levels <50 copies/mL at week 48 and no confirmed virologic failures in the DTG + FTC group [30]. Treatment optimization in the SIMPL’HIV study also involved the evaluation of a simplified patient-centered monitoring (PCM) strategy vs standard routine monitoring (SM). Here, we present long-term results from the 144-week analyses of the SIMPL’HIV study.

## METHODS

### Study Design and Participants

The SIMPL’HIV study was conducted at 7 Swiss HIV Cohort Study sites [33], enrolling adults (≥18 years) on EACS-recommended cART who were virologically suppressed for at least 24 weeks. Exclusions included previous treatment change due to unsatisfactory response, integrase strand transfer inhibitor (InSTI) resistance mutations, pregnancy, HIV-2 infection, renal and liver impairment, hypersensitivity to DTG or FTC, nonadherence, and active hepatitis B infection. A protocol amendment allowed inclusion of patients with the M184V mutation beginning June 27, 2017. Written informed consent was obtained, and the study was conducted according to ethical guidelines.

### Procedures and Randomization

Participants were randomized 1:1 to switch to DTG + FTC or continue cART and to PCM or SM. SM involved trimonthly laboratory tests and in-person visits at Swiss HIV Cohort Study sites. PCM included tests only at weeks 0 and 48, with personalized options for phone consultations, home or work drug delivery, and blood tests at decentralized certified private laboratories. An independent statistician generated a stratified random allocation sequence using a computer-based system, employing randomly permutated block sizes 4 and 8 to ensure balanced randomization by study site.

At week 48, participants could change their antiretroviral regimen based on shared decision-making with their physician and were given the possibility to select 1 or more options of PCM, according to local availability. HIV-1 RNA load, immunology, and safety measures were assessed at weeks 96 and 144. Weight, adverse events (AEs), medications, adherence, quality of life, and satisfaction (measured using the PROQOL-HIV questionnaire and visual analog scales) were recorded at each visit until week 144 [34].

### Outcomes

The main end point was the proportion of participants with HIV-1 RNA <100 copies/mL through 144 weeks. Secondary end points included the proportion with HIV-1 RNA <50 copies/mL at weeks 96 and 144, AEs, changes in CD4 count, lipid profile, glucose, renal and hepatic function, weight, quality of life, and patient satisfaction between baseline and week 144.

### Statistical Analyses

The main end point was assessed in the intention-to-treat (ITT) population. The statistical analysis plan prespecified analysis at weeks 96 and 144; however, a defined margin of noninferiority was only established for the primary outcome at the week 48 analysis. Binary end points were analyzed using Cochran-Mantel-Haenszel statistics; continuous variables were analyzed with linear regression. All analyses were performed using R software, version 3.6.1, following CONSORT guidelines for noninferiority trials [35].

## RESULTS

### Participants

**Figure 1.**
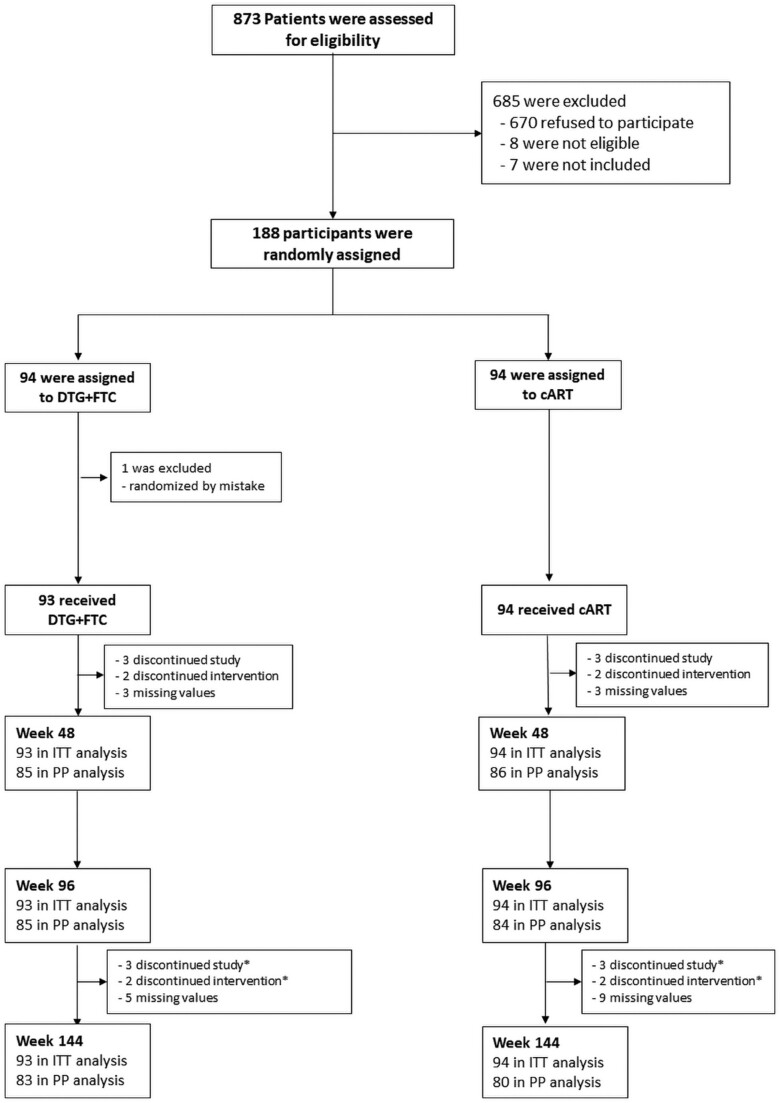
CONSORT flowchart of recruited patients. ^a^Participants discontinued the study between baseline and week 48. Abbreviations: cART, combined antiretroviral therapy; CONSORT, Consolidated Standards of Reporting Trials; DTG, dolutegravir; FTC, emtricitabine; ITT, intention-to-treat; PP, per-protocol.

Screening for the SIMPL’HIV study began on January 1, 2018, and concluded with the last participant visit on May 17, 2021. Out of 873 individuals screened who met inclusion criteria, 188 participants were randomly assigned to either the DTG + FTC group (93 participants) or to continue cART (94 participants). One randomization error led to an ineligible participant being included (Figure 1). Baseline characteristics were comparable between the groups, as detailed in the article reporting 48-week results [30].

### Efficacy

In the ITT analysis at week 144, 87.1% of participants initially assigned to DTG + FTC group and 81.9% of those initially randomized to the cART group maintained HIV-1 RNA levels <100 copies/mL, demonstrating comparable efficacy (adjusted difference, 5.2%; 95% CI, −5.3% to 15.4%). According to the FDA algorithm, 86% of participants in the DTG + FTC group and 79.8% of patients in the cART group had HIV-1 RNA <50 copies/mL at week 144 (adjusted difference, 6.2%; 95% CI, −4.7% to 16.8%). One patient in the DTG + FTC group and 4 in the cART group had HIV-1 RNA levels >50 copies/mL at week 144. By week 96, virological suppression rates were comparable in the treatment groups, at 88.2% in the DTG + FTC group and 84% in the cART group. The mean CD4 gain between baseline and week 144 was 19.7 (±175.8) and 35.6 (±195.1) cell/mm^3^ with DTG + FTC and cART, respectively (adjusted difference, −11.8; 95% CI, −67 to 43.4). Data were missing for 19 participants, and follow-up was lost for 6.

#### Safety

The incidence of AEs was similar between groups (DTG + FTC, 73.1%; cART, 64.9%; adjusted difference, 8.3%; 95% CI, −5.1% to 21.4%). Common AEs included upper respiratory infections, headaches, and diarrhea. Serious AEs (SAEs) were more common in the cART group (18.1%) compared with the DTG + FTC group (12.9%). SAEs were not related to the study medications. One participant in each arm had an AE leading to discontinuation of the study drug (suicidal ideation in the DTG + FTC arm at week 6 and arthralgia in the cART arm at week 36). Weight increased in 62.4% of DTG + FTC and 59.6% of cART participants, with similar proportions gaining ≥10% of their body weight. No significant differences were found in renal, lipid, or glucose profiles (Table 1).

**Table 1.** Safety Outcomes: Changes Between Baseline and Weeks 96 and 144^a^

Safety End Point	DTG + FTC (n = 85)	cART (n = 86)	Adjusted Risk Difference (95% CI)	*P* Value
CD4 count (week 96), cells/mm^3^	24.3 (±178.5)	30.0 (±216.7)	0.5 (−60.5 to 61.5)	.987
CD4 count (week 144), cells/mm^3^	19.7 (±175.8)	35.6 (±195.1)	−11.8 (−67.0 to 43.4)	.673
Lipidic profile	…	…	…	
Total cholesterol (week 96), mmol/L	−0.3 (±0.8)	−0.1 (±0.8)	−0.1 (−0.3 to 0.2)	.556
Total cholesterol (week 144), mmol/L	−0.2 (±0.8)	−0.1 (±0.7)	−0.1 (−0.3 to 0.1)	.396
LDL (week 96), mmol/L	−0.3 (±0.9)	0.0 (±0.6)	−0.2 (−0.5 to 0.0)	.045
LDL (week 144), mmol/L	−0.2 (±0.8)	0.1 (±0.7)	−0.2 (−0.5 to 0.0)	.070
Creatinine (week 96), mmol/L	0.5 (±10.2)	0.1 (±13.7)	0.5 (−3.2 to 4.3)	.790
Creatinine (week 144), mmol/L	1.3 (±11.0)	0.4 (±11.7)	0.7 (−2.9 to 4.3)	.690
Glucose (week 96), mmol/L	−0.1 (±1.2)	0.1 (±1.4)	−0.2 (−0.5 to 0.1)	.273
Glucose (week 144), mmol/L	0.3 (±2.2)	0.0 (±1.4)	0.3 (−0.3 to 0.8)	.341
Weight (week 96), kg	1.6 (±4.3)	1.7 (±4.0)	−0.1 (−1.4 to 1.2)	.834
Weight (week 144), kg	2.3 (±4.7)	2.6 (±3.9)	−0.2 (−1.6 to 1.1)	.729

Abbreviations: cART, combined antiretroviral treatment; DTG, dolutegravir; FTC, emtricitabine; LDL, low-density lipoprotein.

^a^Outcomes are reported with mean ± SD.

#### Quality of Life and Patient Satisfaction

At week 144, quality of life scores, measured using the PROQOL-HIV questionnaire, were high, with scores consistently >80/100 points across all participants. Patient satisfaction with monitoring and treatment remained high throughout the study, with no significant differences between treatment groups.

#### Study Options

During the poststudy period, participants could initiate or stop alternative monitoring options. Call visits were initiated by 75 (40%) participants at baseline and continued by 58 (31%) at week 144; drug delivery was chosen by 48 (26%) individuals at baseline and continued by 40 (21%) at week 144; 17 (9%) participants performed blood tests in an alternative laboratory at baseline, and 23 (12%) confirmed this choice at week 144. At week 144, 83.1% of participants on DTG + FTC and 26.8% of those in the cART group opted to remain on dual therapy, switching to the DTG/3TC single-pill formulation available on the market.

## DISCUSSION

The SIMPL’HIV study, a nationwide academic-led trial conducted across Switzerland, demonstrated the efficacy and safety of DTG + FTC as a 2-drug regimen compared with cART in people with HIV-1 infection at week 48. At the end of the randomization period at week 48, participants were offered the choice of dual vs standard cART. The vast majority of patients assigned to dual therapy choose to keep this option, while 26% only of those on cART decided to switch to the dual therapy of DTG + FTC. At 144 weeks, 87.1% of participants originally assigned to DTG + FTC and 81.9% of those assigned to cART had HIV-1 RNA levels <100 copies/mL.

While no specific clinical trial has evaluated the efficacy and safety of DTG + FTC as a 2-drug regimen, existing studies on similar combinations can provide valuable context. FTC shares similarities with 3TC in terms of convenience, safety, and resistance profile. However, FTC boasts a longer intracellular half-life and demonstrates greater in vitro potency [36], rendering it a favorable choice for the SIMPL’HIV trial at the time the protocol was written in 2016. However, the commercialization of a co-formulated version of DTG/3TC in 2019 made a DTG + FTC regimen a less favorable option.

Our findings are consistent with the industry-led TANGO trial, in which 86% of patients had virological suppression in the DTG/3TC arm vs 82% in the standard ART arm at week 144 [11]. However, SIMPL’HIV had few eligibility restrictions and included a more diverse participant population (no restriction in the comparator cART arm, any US Centers for Disease Control and Prevention clinical stage, nadir CD4 count, HIV-1 zenith viral load value, or M184V mutation). Interestingly, the applicability of DTG-based dual therapy has enlarged to larger groups of patients, and Blick et al. [32] demonstrated the efficacy up to week 144 of a similar regimen in patients harboring a M184V mutation. The low rate of virologic failure observed through 144 weeks in SIMPL’HIV is consistent with findings from a meta-analysis that found that DTG/3TC as maintenance therapy resulted in virologic failure in only 1% of treatment-experienced patients at either week 48 or week 96 [37].

Safety analyses in SIMPL’HIV showed stable CD4+ cell counts and similar rates of AEs and weight gain between the DTG + FTC and cART arms, aligning with long-term findings from the TANGO trial [11]. The study also evaluated alternative monitoring options, such as call visits, drug delivery, and laboratory tests at alternative locations, which were well received by participants. However, these options did not reduce costs compared with standard monitoring [38].

The study had some limitations, including a predominantly White, male, and young cohort, and potential underreporting of adverse events due to extended follow-up intervals and COVID-19 disruptions.

The SIMPL’HIV study supports DTG + FTC as an effective and durable maintenance therapy for HIV-1 infection. It demonstrates comparable efficacy to 3-drug regimens and offers a viable alternative with a good safety profile and high patient satisfaction, paving the way for broader adoption of this 2-drug regimen in HIV management.

## Notes

***Author contributions.*** All authors had full access to the data and have provided their final approval of the version of the manuscript to be submitted for publication.

***Clinical Trials registration.*** NCT03160105.

***Availability of data and materials.*** Granting access to this information will be evaluated on a case-by-case basis upon reasonable request. Data access should be requested to the first author.

***Financial support*.** This study was financed within the framework of the Swiss National Science Foundation (grants # 166819, 17481) and The Swiss HIV Cohort Study (SHCS), supported by the Swiss National Science Foundation (grant #177499), by SHCS project #826, and by the SHCS research foundation. A.C. was in receipt of grants.
